# Supplementary material for: Malnutrition among under-five children in amhara and oromia regions, Ethiopia: Continuous time markov multi-state modeling
Source: PLoS One. 2025 Aug 21;20(8):e0330537. doi: 10.1371/journal.pone.0330537 (PMC12370122; doi:10.1371/journal.pone.0330537)
Supplement: S1 Table — (PDF) [file pone.0330537.s001.pdf]

Supporting Information

S1 Table: Prevalence of Malnutrition among Under-Five Children

Variables	Categories	Rounds					
		Baseline		Midline		Endline	
		Count	%	Count	%	Count	%
Stunting status of U5C	Stunted	1140	37.5%	1560	52.5%	1292	46.0%
	Normal	1733	56.9%	1313	44.2%	1481	52.6%
	Tall	171	5.6%	100	3.3%	40	1.4%
	Total	3044	100.0%	2973	100.0%	2813	100.0%
wasting status of U5C	Wasted	409	13.5%	185	6.2%	240	8.7%
	Normal	2185	72.2%	2511	84.7%	2308	83.5%
	Overweight	432	14.3%	269	9.1%	216	7.8%
	Total	3026	100.0%	2965	100.0%	2764	100.0%
Underweight status of U5C	Under-weight	723	23.8%	811	27.3%	835	29.7%
	Normal	2051	67.4%	2024	68.1%	1917	68.1%
	Overweight	270	8.9%	138	4.6%	61	2.2%
	Total	3044	100.0%	2973	100.0%	2813	100.0%
