# Supplementary material for: Malnutrition among under-five children in amhara and oromia regions, Ethiopia: Continuous time markov multi-state modeling
Source: PLoS One. 2025 Aug 21;20(8):e0330537. doi: 10.1371/journal.pone.0330537 (PMC12370122; doi:10.1371/journal.pone.0330537)
Supplement: Attachment — Submitted filename: Response to Reviewers.docx [file pone.0330537.s003.docx]

Response to Reviewers Comments

Journal: PLOS ONE

Manuscript: Ref: Submission ID: **PONE-D-24-37812**

Title: Malnutrition of Under-Five Children in Amhara and Oromia Regions, Ethiopia: Continuous Time Markov Multi-State Modeling

### Dafa Duge Wachifo, Dereje Danbe and Zeytu Gashaw Asfaw

Dear Editor,

We are grateful to you and the reviewers for taking the time to read our article and offer insightful criticism. The current version may be improved as a result of your insightful and important feedback. The authors have given the feedback great thought, and we have done our best to respond to each and every one. The entire section of the manuscripts has been read through, and a careful revision has been undertaken. We now anticipate a significant improvement in the overall writing quality and readability. Each reviewer issue has been thoroughly read and corrected, rewritten, or rephrased. Our point by point responses are enclosed below.

**The point by point response**

**Journal Requirements:**

1. When submitting your revision, we need you to address these additional requirements. Please ensure that your manuscript meets PLOS ONE's style requirements, including those for file naming. The PLOS ONE style templates can be found at
   <https://journals.plos.org/plosone/s/file?id=wjVg/PLOSOne_formatting_sample_main_body.pdf> and 
   <https://journals.plos.org/plosone/s/file?id=ba62/PLOSOne_formatting_sample_title_authors_affiliations.pdf>

- **Author Response:** We have revised based on PLOS ONE’S style as indicated in both main body and Title page guidelines.

2. When completing the data availability statement of the submission form, you indicated that you will make your data available on acceptance. We strongly recommend all authors decide on a data sharing plan before acceptance, as the process can be lengthy and hold up publication timelines. Please note that, though access restrictions are acceptable now, your entire data will need to be made freely accessible if your manuscript is accepted for publication. This policy applies to all data except where public deposition would breach compliance with the protocol approved by your research ethics board. If you are unable to adhere to our open data policy, please kindly revise your statement to explain your reasoning and we will seek the editor's input on an exemption. Please be assured that, once you have provided your new statement, the assessment of your exemption will not hold up the peer review process.

- **Author Response:** we all agreed to make data available on acceptance of manuscript.

3. Your ethics statement should only appear in the Methods section of your manuscript. If your ethics statement is written in any section besides the Methods, please move it to the Methods section and delete it from any other section. Please ensure that your ethics statement is included in your manuscript, as the ethics statement entered into the online submission form will not be published alongside your manuscript.

- **Author Response**: Thank you very much for suggestion we did it as commented.

**Additional Editor Comments**

1. **The background section of the article needs to be modified.** 1) Certain repetitive expressions should be simplified, such as the harm of under-nutrition to children's growth and development.

**Author Response:** we tried to simplify them and have revised it by rewriting as presented page 2 in track change file.

2) Please simplifying and merging the first and second paragraphs into one paragraph in the background.

**Author Response:** very good suggestion, we have revised it by rewriting and merging paragraph 1, 2 and 3 and presented in page 2 & 3 of track change file.

3) The website is not recommended to be shown in the third paragraph and can be used as a reference.

**Author Response:** Good comment, we have improved it by substituting reference with meeting minute report of five UN agencies (Mitchell & Catherine, 2023).

4) Please explain the statement “The CIAF of children has a transient nature, which needs to be addressed using a multi-state model.”

- **Author Response:** we revised it by elaborating like: The CIAF of children reflects a transient or dynamic nature which needs to be analyzed using a multi-state model. That is, children’s nutritional status can change over time due to a variety of factors such as dietary changes or intervention, health condition, natural and manmade random shocks (drought, locust infestation, pandemic, and war so on). CIAF is composite measure that aggregates the different status of malnutrition (stunting, wasting, underweight and over-weight) and can be used to track the overall malnutrition. A child’s status can move from one CIAF state to another or recovery back healthy state, making it a dynamic. Thus multi-state model allows for understanding of how children might move between different states of CIAF ( under failure, healthy, over failure or remission to these states).

5) I don't quite understand the theoretical and practical basis of this study. The author listed many studies in detail in the last section, but it seems that the connection to the theme of this study is not so direct. I personally strongly agree with the author's viewpoint of that more research is required to determine the effects of improved food diversity on children's nutritional status and to examine the intervention's consequences. However, this study did not directly address this issue. Please elaborate on the theoretical and practical basis that is more directly related to the purpose of this research. For example, regarding the topic of changes in nutritional status and influencing factors, what problems have been solved in previous research, what problems have not been solved, and what social significance does solving this problem have.

- **Authors Response:** Thank you for comments and question asked for, we tried to revise based on question and presented at page 3 to 5 in track change file as **“**Previous studies have attempted to identify determinants of under-nutrition using anthropometric measures such as stunting, wasting or underweight separately, but they neither aggregate the indexes nor consider its transient or dynamic nature ( Mohammed & Asfaw, 2018; Amare et al., 2019; Fenta et al., 2020; Belay et al., 2023; Bitew et al., 2021; Raru et al., 2022). Comparing stunting, under-weight, and wasting separately cannot provide a single conclusion on the overall burden of malnutrition among children. Composite index anthropometry failure (CIAF) is preferable to address malnutrition, as WHO criteria and Waterlow's classification which do not discriminate between distinct conditions of indices of malnutrition, (Kuiti & Bose, 2018). The studies conducted by (Fenta et al., 2021a; Kassie & Workie, 2020) in Ethiopia tried to indentify determinants of under-nutrition using composite index; but they did not consider dynamic characteristics of the response nor include overnutrition in their composite index. More recently, Oduro et al., (2024) had studied dynamic characteristics of stunting by using longitudinal data at Nairobi, Kenya and applied multi-state model but they did not include other indicators of malnutrition. As to authors’ knowledge, the studies conducted so far have not fully integrated CIAF in a way that it includes neither under-nutrition and over-nutrition components, nor the dynamic nature of the malnutrition.

2. **Methods section:** 1) Please show some details about the process of sampling.

**Author Response**: very good suggestion, were revised as “initially, 196 kebeles from Amhara (115 kebeles) and Oromia (81 kebeles) were selected from 15 productive safety net program (PSNP) implemented districts. However, four kebeles (3 from Amhara and 1 from Omromia) regions were dropped due to two kebeles had no PSNP clients and two kebeles were experienced ongoing civil unrest. Thus, the evaluation sample comprises 192 kebeles. From each kebele 18 households were randomly sampled and final samples those satisfied inclusion criteria were 3,314 households. The inclusion criteria for the sample were that households had to be a PSNP client household, had to have at least one child aged 0–35 months at baseline to have child with under-five age after 2 years follow up, and had to have the mother or primary female caregiver of the 0–35-month-old child as a member of the household.”

2) I am very confused about what the authors want to say by citing many references when describing the independent variables. Is the selection of independent variables based on these references, or are the definitions of these independent variables referenced from these literature sources?

**Author Response:** NO. Definitely right question and comment; it was technical error of author we revised it by removing all references listed there.

3) “Please describe the specific version of the statistical software, including R and SPSS. You should even specify which R package program was used.”

- **Author Response:** very good suggestions, we revised it like: Data clearance and analysis were performed by SPSS version 27, and R version 4.3.2 with msm 1.7.1. The standard scores of all anthropometric measure indexes were computed by zscorer 0.3.1 packages.

6) The results section needs to be streamlined and reorganized. 1) There are so many tables and figs shown in the results section, so that the result appears to be without focus. Here are my some suggestions. Table 2 can be presented as an appendix and briefly described in the main text. The results section should present the basic characteristics of the sample at first to the readers. Table 4 and Table 5 can be merged into one table. Figures 3 and 4 provide too little information for readers. In other words, readers cannot interpret useful information from the Figures. Delete these two figs. If the authors think they are very important, they can be attached in the appendix.

- **Author response:** We accept all editor suggestions as they are because we are agree to minimize number of Tables and figures from main body of manuscript without sacrificing much information. Since, Tables 4 & 5 are revealed the same output but author had separated it in to two as Table 4 and Table 5 to avoid long Table which is appearing more than one page. So, it can be merged in to one as suggested. Again Figures 3-5 indicate that the movements of children’s nutritional status (CIAF) among 3 specific states. They showed that there were transitions from each state or transitions are bidirectional. That is specific child can transits forward or backward among any of 3 states during study period. Their difference was number of children in process. For instance, in Figure 3, we had plotted transitions of sampled children all together, in Figure 4, we had plotted male and female separately and in Figure 5, we had plotted 4 unique selected children. Therefore, if we delete Figures 3 & 4 and retain Figure 5 as suggested by editor; It is very good insight, here we will benefit with précising the idea. Hence, all suggestions were accepted and improved as shown in track change file from page 9-14.

2) The description of Figure 5 needs to be modified. What kind of result does Figure 5 want to express.

- **Authors Response:** We have modified the interpretation of Figure 5 and presented in track change file page 14. It shows the same things as Figure 3 & 4 but transitions in it were more clear and simple to visualize since sample is small. It indicates that the transition of nutritional status (CIAF) of children or its dynamic characteristics that is it changes as time passes. For example, a child in normal only state at time t can transit to either under failure state or over failure state or stay at normal only state at t+1.

3) In the table 4, "Spouse or partner is a member of the hh?", what's meaning for 'hh'?

- **Authors Response:** In Table 4, “Spouse or partner is a member of the hh?” question is right we accepted comment and modified it as “Spouse or partner is a member of the household (hh)?”

4) I'm not sure what kind of result the author wants to describe in Figure 6? Many of the results statement described are the author's speculations and should not be included in the results. The description of the results should be objective.

- **Authors Response:** We have modified the description of Figure 6 as it, revealed that whether the multi-state model is good fit to the data or not. Based on the result the estimated prevalence rate and observed prevalence approximately equal. Hence, model is good fit to the data.

5) The results of Table 10 are difficult to be understood. For example, a child at normal only state has probability 0.71 being in under-nutrition in the next , and 0.29 being in over-failure, What is the probability of the state remaining unchanged? The author should provide the total probability of transitioning to the next state (not including unchanged state).

- **Authors Response:** Table 10, “….The author should provide the total probability of transitioning to the next state (not including unchanged state).” Really, we appreciate editor’s view and question on probability of remain unchanged in the same state. But the theoretical basis for probability in next state is different from probability of transition. Its aim is to answer the question that, if movement of subject/object is must then where he/she is more probable to move? In our case we know that specific child had transition from health state then we want to estimate how more probable he/she transit to either of states. That is healthy child is more probable (0.71) to transit under failure state than over failure state.

**Reviewer #1**

Thanks for the opportunity to review this manuscript. The study analyzes transition rates, durations, and factors influencing the movement between different nutrition states using data from three survey rounds conducted by the International Food Policy Research Institute. I suggest to put multi state among the keywords

1. **Abstract:** In the method section there is a minor grammatical issue in "The institute was performed the three consecutive follow-up surveys rounds." Consider revising to "The institute conducted three consecutive follow-up survey rounds." The results section is detailed, but some of the findings could be presented more concisely. For example, the phrase "The results indicated that the probability being in the under-failure state in the next time for a healthy infant is higher (0.71)" could be revised to "The probability of a healthy infant transitioning to the under-failure state is high (0.71). "

**Author Response:** Thank you very much for your valuable suggestions, we tried to modify each of them. At the beginning we included “Multi-State Model” among key words but latter author excluded after reading general guideline for key words which suggests that not to include words in the title. The guideline that I have read may be view of scholars so; I am happy to include “Multi-State Model” in key words and also we have revised suggestions to improve the abstract.

2. **Introduction:** The introduction is lengthy and could benefit from being more concise. Consider breaking it into shorter paragraphs to enhance readability and separating the background information from the study's Objectives: There are repeated references to data from UNICEF, WHO, and other organizations. Condense the references to these organizations by summarizing the main points.

- The study's objective is introduced at the end of a very long paragraph. Consider moving it earlier for better context and rephrasing for simplicity.

- **Author Response:** very good suggestions; we tried to improve them by separating background information into paragraph1 and objectives into paragraph2 at same time tried to minimize reference repletion by summarizing points.

3. **Research method**: 1) The detailed description of the IFPRI data collection process, including the virtual meeting dates and contacting procedures could be simplified

**Author Response:** thank you for comment, we revised by moving statement in to last part of next paragraph at page 6 &7 in track change file and simplified as: “By presenting brief objectives of the current study, the authors contacted and requested IFPRI to get access of the data set. Finally, the data set was accessed on May 29, 2023.”

2) The section describing the inclusion criteria and sample selection is clear but could be streamlined for readability.

**Author Response:** Thank you for suggestion, we tried to revise it as presented in track change file page 7: “The inclusion criteria for the sample were that households had to be a PSNP client household, had to have at least one child aged 0–35 months at baseline to have child with under-five age after 2 years follow up, and had to have the mother or primary female caregiver of the 0–35-month-old child as a member of the household.”

3) The description of the Multi-state Markov Model is technically dense. Consider breaking it into smaller paragraphs with simple explanations before diving into the complex equations.

**Author Response:** Good suggestion, we tried to revise by dividing in to paragraphs and defining some subscripts as shown in page 11 and 12 in track change file.

4) Under independent variables, provide a brief rationale for why each variable was selected. This will highlight their relevance to the study.

**Author Response:** Thank you very much for your suggestion; we revised the rationale of including each covariate as “The explanatory variables included were based on aim of study, their availability in data set used and recommended as significant predictors of child malnutrition in literatures. The aim of this study was estimating metrics of transition of nutritional status of U5C. Thus the inclusion of covariates was not exhaustive. As per these reasons, the covariates proposed for this study were as follows:”

Equations: The equations are not consistently formatted and use a mixture of symbols, parentheses, and notation that makes them hard to follow. 5) Some symbols are not clearly defined before they are used. For instance, " ℎ ( )" appears in the equations without a prior explanation of what each component represents. - The text mentions " ( )" as part of the multi-state model but does not define what " " specifically represents (e.g., the state of the child's nutritional status).

- The equation “(3)” is introduced, but there seems to be a mismatch between the equation numbering and the accompanying text, making it confusing to follow the derivation or steps.
- Each equation is presented without sufficient explanation of its purpose or the meaning of the symbols.
- When equations are introduced, provide a sentence that connects the mathematical expression to the research context (e.g., how the transition rates are used in analyzing children’s nutritional states).

**Author Response:** Thank you very much for your valuable suggestions in model part; we tried each of them and improved them by describing equations, defining subscripts –superscripts, symbols and adjusting numbering of equation as shown on page 10 to 14 in track change file.

4. Results: I suggest to Move the interpretation of key findings (e.g., transition probabilities, covariate effects) into the discussion section, where you can explore the implications more thoroughly.

**Author Response:** We have revised as suggested: by taking interpretations of key finding to discussion part in manner which it shows implication of the result.

-The statement "Indexes in all rounds were much greater than the current national estimates" is valuable but would benefit from specifying what the national estimates are for comparison.

**Author Response:** We had revised moving this statement to discussion on prevalence of malnutrition section and like : Current national estimates were 34.4% of stunting, 6.8% of wasting and 21% of underweight prevalence (UNICEF et al., 2023)

-For each table, provide a brief, clear interpretation of its data. For example,
- While the results provide detailed statistical outputs, there is a need to better link these findings back to the study's objectives. For example, the study aims to understand the transition rates, durations, and drivers of under-nutrition states. Explicitly stating how the results address these goals would clarify the narrative and emphasize the study's contributions.

**Author Response:** Thank you very much for very good suggestion, we tried to revise interpretation and connected it with study’s objective as indicated in track change file.

5. Discussion: Organize the discussion around key themes or findings, such as the prevalence of malnutrition, transition probabilities, and the influence of covariates. This structure will help readers easily grasp the main points

**Author Response**: Very good suggestions, we improved as suggested

- the finding that female children are less likely to transition to under-nutrition is mentioned but not fully explained. Including possible reasons, such as differences in care or cultural practices, would provide more context and depth.

**Author Response:** Very good suggestions, we improved as: This finding might be due to cultural and genetic factors (maternal and newborn factors or endocrine/immune factors). Culturally, the gender perception might be one of the factors; in Ethiopia’s case boys are prioritized for the superior option. So, boys receive complementary food earlier than girls because breast-milk is viewed as inferior to complementary foods. This might increase the risk of infection or malnutrition, especially if hygiene standards are not met. In addition, boys are viewed as heroes who are allowed to play freely outside the home might also contribute to increased exposure to infections or risky situations. Boys being more mobile and having greater freedom might encounter more opportunities for germs, especially if sanitation practices are inadequate. These infections can disrupt the body's nutrient absorption, exacerbating malnutrition. Furthermore, the sex difference which states that male placentas are smaller by ratio to birth-weight to accommodate a larger foetus in the womb and so male placentas potentially have reduced reserve capacity. This accelerated growth may be beneficial in the presence of an abundant food supply but this makes males vulnerable to food shortages, (Eriksson et al, 2010, Thurstan et al 2022). This problem is more pronounced among lower socio-economic groups and at higher levels of food insecurity (Wamani et al, 2007). A stronger immune response and capacity to produce antibodies in girls than boys and the interaction between sex hormones and environmental factors has consequences for energy consumption, nutritional requirements and vulnerability to infectious diseases, (Thurstan et al, 2022).

- The explanation of probabilities and transition rates is often overly technical. While it's important to be precise, translating these findings into real-world implications (e.g., the risk and duration of under-nutrition in children) would make the discussion more accessible and impactful.

**Author Response:** Very good comment, we tried to improve as suggested.

- the statement, "This result is consistent with (Oduro et al., 2024) but it is in contrast with (Häkkänen et al., 2020; Hu et al., 2022; Moreira et al., 2019)" does not explain why there might be differences between these studies. Discussing possible reasons for these inconsistencies.

**Author Response:** Very good comment, we improved as: The current study and the study by Oduro et al were from neighboring countries or sub-Saharan Africa countries. Hence, there might be similarities in socioeconomic status, dietary habits, and healthcare infrastructure in these neighboring countries; so, there is consistency in the finding. However, the studies by Häkkänen et al, Hu et al and Moreira et al were from developed countries; the reason for inconsistency in the finding with these studies might be major malnutrition problem in developed countries was over-nutrition. The causes, risk factors, and even the public health responses to malnutrition can vary significantly.

- The discussion section focuses heavily on the Composite Index of Anthropometric Failure (CIAF) rather than exploring the full potential and unique insights provided by the multi-state model. While the CIAF is an essential part of the study, the use of a multi-state Markov model is one of the study's most novel and complex aspects, and it deserves more thorough discussion.

**Author Response:** Thank you for your valuable comments; we revised in discussion section at sub-section strength of the study like: Since, the response exhibits a transient or dynamic nature. To appropriately estimate the varying risks of transition and their associated covariate effects, the study employed a continuous-time Markov multistate model. This approach is rarely applied in the field of nutrition, making it a novel contribution of the current study. The finding showed that the model provided a good fit to the data, as evidenced by the comparison criteria used.

**Reviewer #2**

1. In the introduction section, it is explained that Ethiopia faces high burden of under-nutrition prevalence, ranking among 15 worst affected nations, perhaps short-term or long-term targets in that field in Ethiopia can be explained as a reference.
   While the prevalence of malnutrition in Ethiopia is discussed, could comparisons to neighboring countries or regions add further perspective to the problem?

- **Author Response:** Thank you very much for the question; Ethiopia is one of among globally affected countries but most of listed 15 countries (Afghanistan, Burkina Faso, Chad, DRC, Ethiopia, Haiti, Kenya, Madagascar, Mali, Niger, Nigeria, Somalia, South Sudan, Sudan and Yemen) were from Africa except Haiti, Afghanistan and Yemen.

2. The methodology mentions anthropometric measurements as a key data source. How were these measurements standardized across different survey rounds, and what steps were taken to minimize measurement errors?

- **Author Response:** Thank you for good question. We revised it as to minimize measurement errors of the response, at time of the data collection every child’s and mother’s / primary female care giver’s height/length, middle upper arm circumference (MUAC) and weight were measured twice by trained anthropometric enumerators. If the differences between the two measurements for height/length and MUAC were less than 0.01cm and 0.01Kg for weight then considered as accurate to record. If the difference was greater than aforementioned values then third measurement had taken and recorded. The standard scores were computed by using zscore *R package* for each malnutrition indicators: height-for-age standard score (HAZ), weight-for height standard score (WHZ) and weight-for-age standard score (WAZ).

3. The study uses a continuous time Markov multistate model. Could the authors briefly describe why this model was chosen over other statistical models?

**Author Response:** In time to event data, Kaplan–Meier estimator and Cox proportional hazard models are adequate to use in studies where there is only one type of event of primary interest. The competing risks models can accommodate transition between one initial state and several mutually exclusive absorbing states. The recurrent event models are appropriate when transition from one state to another state, either recurrent event 1 or event 2 or so on. However, when there are multiple events of interest; these methods may not provide a full picture of the relationship. In such case multistate model provides a flexible and broader framework to extend familiar methods. When the process involves transitions between several well defined distinct states in such case multistate model is appropriate over other statistical models (Le-Rademacher, 2022). The progression of nutritional status or CIAF of U5C is an example of complex processes with intermediate events. Therefore, multistate model is preferred over other statistical model in this specific study.

4. The results indicate that children under 5 spend 75% of their time in under-failure states. Could the authors elaborate on why recovery times are so long and suggest factors that may contribute to this?

**Author Response:** The households included in the study were poorest and who were eligible to be the member of PSNP. This finding might be attributed to the civil war in the country during the data collection period, the locust infestation, and the zones included in this study were also severely impacted by recurring drought. These combined shocks likely have a greater impact on child nutritional status stay longer in under failure state.

In the results section, it is explained that Girl had 1.824 times higher likelihood of recovering from an over failure state and less likely to go from a healthy state to an under-failure state compared to boy, perhaps the factors influencing this can be mentioned.

**Author Response:** Very good suggestions, we improved as: This finding might be due to cultural and genetic factors (maternal and newborn factors or endocrine/immune factors). Culturally, the gender perception might be one of the factors; in Ethiopia’s case boys are prioritized for the superior option. So, boys receive complementary food earlier than girls because breast-milk is viewed as inferior to complementary foods. This might increase the risk of infection or malnutrition, especially if hygiene standards are not met. In addition, boys are viewed as heroes who are allowed to play freely outside the home might also contribute to increased exposure to infections or risky situations. Boys being more mobile and having greater freedom might encounter more opportunities for germs, especially if sanitation practices are inadequate. These infections can disrupt the body's nutrient absorption, exacerbating malnutrition. Genetic factors, the sex difference which states that male placentas are smaller by ratio to birth-weight to accommodate a larger foetus in the womb and so male placentas potentially have reduced reserve capacity. This accelerated growth may be beneficial in the presence of an abundant food supply but this makes males vulnerable to food shortages, (Eriksson et al, 2010, Thurstan et al 2022). This problem is more pronounced among lower socio-economic groups and at higher levels of food insecurity (Wamani et al, 2007). A stronger immune response and capacity to produce antibodies in girls than boys and the interaction between sex hormones and environmental factors has consequences for energy consumption, nutritional requirements and vulnerability to infectious diseases, (Thurstan et al, 2022).

5. The authors can strengthen the practical recommendation section by elaborating on specific interventions for policymakers. For example, what types of maternal education programs would be most effective?

- **Author Response:** good question, we revised it as: Government and NGO could plan for adult education programs to improve maternal knowledge and skill on child nutrition, diet diversity, child care, hygiene, family planning and the like that improve child nutrition in rural community of the country and to shift cultural attitudes about gender roles and their impact on well-being.

6. In the discussion section, it is explained that the child being live in Oromia region is less likely transit from healthy state to malnutrition as compared with child in Amhara region, perhaps the factors influencing this can be mentioned.

- **Author Response:** thank you for good comment, we revised like: This finding might be attributed to the war in northern Ethiopia during the data collection period, the locust infestation, and the zones included in this study from Amhara, which were also severely impacted by recurring drought. These combined shocks likely have a greater impact on child nutritional status in Amhara compared to Oromia.

7. Considering the long recovery periods highlighted in the study, could the authors propose emergency interventions to prevent children from remaining in under-failure states for extended periods?

- **Author Response:** thank you for the question, we would understood question, it is revised as: GO/NGO could plan for special intervention strategies on random shocks like recurring drought, locust infestation, conflicts to reduce the duration and impact of under failure states among U5C.

8. In the discussion section, related to the limitations mentioned, it is possible to explain recommendations for further research so as not to encounter similar limitations that can be in line with the recommendations from the research results in the conclusion section.

- **Author Response:** Thank you very much for good comment; we tried to improve avoiding redundancy of suggested statement from limitation section and considering it in recommendation section only.
