# Supplementary material for: Malnutrition among under-five children in amhara and oromia regions, Ethiopia: Continuous time markov multi-state modeling
Source: PLoS One. 2025 Aug 21;20(8):e0330537. doi: 10.1371/journal.pone.0330537 (PMC12370122; doi:10.1371/journal.pone.0330537)
Supplement: Attachment — Submitted filename: Response to Reviewers file.docx [file pone.0330537.s004.docx]

Response to Reviewers Comments

Journal: PLOS ONE

Manuscript: Ref: Submission ID: **PONE-D-24-37812**

Title: Malnutrition of Under-Five Children in Amhara and Oromia Regions, Ethiopia: Continuous Time Markov Multi-State Modeling

### Dafa Duge Wachifo, Dereje Danbe and Zeytu Gashaw Asfaw

Dear Editor,

We are grateful to you and the reviewers for taking the time to read our article and offer insightful criticism. The current version may be improved as a result of your insightful and important feedback. The authors have given the feedback great thought, and we have done our best to respond to each and every one. The entire section of the manuscripts has been read through, and a careful revision has been undertaken. We now anticipate a significant improvement in the overall writing quality and readability. Each reviewer issue has been thoroughly read and corrected, rewritten, or rephrased. Our point by point responses are enclosed below.

**ACADEMIC EDITOR:**

**1. Abstract Clarity and Conciseness**

- **Issue**: The abstract is information-dense and includes some awkward phrasing (e.g., “under failure,” “over failure”), which may be unclear to non-specialists.
- **Suggestion**:
- Avoid jargon like “under failure” and “over failure.” Consider using
- Undernutrition or Anthropometric failure, instead of Under failure
- Overnutrition instead of Over failure
- Anthropometric failure (for CIAF) instead of Under failure
- Nourished / No anthropometric failure instead of Normal only
  **Example Rewrite for Clarity:**
- **Original**:
- “Children in the under failure group had higher transition probabilities.”
- **Revised**:
- “Children in the undernutrition group (i.e., those with anthropometric failure) had higher transition probabilities.”
- **Original**:
- “Over failure children had a lower likelihood of reverting to a normal state.”
- **Revised**:
- “Overnourished children (classified as overweight) had a lower likelihood of reverting to a normal nutritional status.”
- Streamline sentences. E.g., *“The probability of a healthy infant transitioning to the under-failure state is high (0.71)”* can be simplified to *“Healthy infants had a 71% probability of becoming undernourished.*

**Authors Response:** Thank you very much for your kind suggestions and insightful comments on the abstract. We have accepted all of them and made the improvements accordingly. Both the track changes and clean versions of the revised file are attached.

**2. Theoretical and Practical Framing**

- **Issue**: The rationale for using the Composite Index of Anthropometric Failure (CIAF) and the Markov model is presented, but the articulation of the *research gap* is buried in repetition and general statements.
- **Suggestion**: Integrate or refer the following relevant articles regarding CIAF

I.Chowdhury M, Billah B, Rashid M, Almroth M, Kader M. Prevalence and factors associated with severe undernutrition among under-5 children in Bangladesh, Pakistan, and Nepal: A comparative study using multilevel analysis. Scientific Reports 2023 Jun 22;13(1):10183.

II.Anik AI, Chowdhury MR, Khan M, Khan TA, Perera NK, Kader M. Urban-rural differences in the risk factors of severe under-5 child undernutrition based on CISAF in Bangladesh. BMC Public Health. 2021 Nov 23;21(1):2147

- Create a sharper distinction between what is already known and what is novel in this study.
- Explicitly state (if so) : *“To our knowledge, no previous study in Ethiopia has used a continuous-time multistate Markov model to analyze both under- and over-nutrition transitions among under-five children using CIAF.”*

**Authors Response:** Thank you very much for your valuable suggestions and comments to improve our manuscript. We have carefully considered and accepted your recommendations. The suggested articles provided us with insightful perspectives, which we used to enhance the quality of our writing and address the identified issues. All revisions have been made and are reflected in both the track-changed (on page 3 at last paragraph) and clean versions of the manuscript.

**3. Methods Section**

- **Strength**: Ethical procedures and data source are well described.
- **Improvement needed**:
- Clarify the logic behind grouping CIAF categories into three states (UF, NO, OF). If possible consider using a flow diagram for clarity.

**Authors Response:** Thank very much again for comment on the logic behind grouping CIAF in to three. The decision to classify children's nutritional status into three broad categories—**under-nourished**, **over-nourished**, and **nourished (normal)**—was guided by the following considerations:

1) **Simplification Based on Nutritional Status:**

Children's nutritional status can be broadly categorized as normal, under-nourished, or o ver-nourished. In our classification, a child was considered *under-nourished* or *over-nourished* if he/she exhibited at least one anthropometric indicator (stunting, wasting, or underweight). That is the corresponding z-score <-2SD and Z-score > +1 SD for over-nutrition. Children not falling into either extreme were considered to have a *normal* or *nourished* status.

2) **Avoiding Complex and Ambiguous Subcategories:**

While it is theoretically possible to define more granular categories—such as **severely under-nourished**, **moderately under-nourished**, **overweight**, and **obese**—this approach introduces several practical challenges. Many children present with overlapping anthropometric failures (e.g., stunting and overweight; wasting and underweight; stunting, wasting, and underweight simultaneously), making it difficult to assign them unambiguously to discrete severity-based categories. Therefore, a more aggregated approach was adopted to reduce complexity and ambiguity.

3) **Modeling Requirements – Application of Multistate Models:**

The use of a **continuous-time multistate Markov model** to analyze the transitions between nutritional states requires a well-defined and manageable number of states. Reducing the classification into three distinct categories facilitates the modeling of dynamic changes in nutritional status over time, ensuring robustness and interpretability of the model outcomes. Graphically, these models are illustrated using diagrams with boxes representing the CIAF states and with arrows presenting possible transition between the states. Here, it was considered the 3-states for child CIAF transition model depicted in Figure 2, and assumed that subjects would be in any given state at time t equal to zero (t=0). The 6 possible transitions were identified by arrows for child CIAF model: 1) from “under-nourished” to “nourished”; 2) from “nourished” to “under-nourished”; 3) from “nourished” to “over-nourished” 4) from “over-nourished to “nourished”, 5) from “under-nourished” to “over-nourished” and 6) from “over-nourished” to “under-nourished”. The child in normal or healthy state at time t can move either to under-nourished (U) state or move to over-nourished (O) state or stay at the same state after time t+1.

Fig 1: Revised and revisited CIAF with nine sections (Kuiti & Bose, 2018)

A=normal status, B=wasting only, C=wasting and underweight, D=stunting + wasting + underweight, E=Stunting + underweight, F=stunting only, Y=underweight only,

G=Stunting + Over-weight, H = Over-weight only

Nourished (N) = A

Under-nourished (U) = B +C + D + E + F+Y

Over-nourished (O) = G + H

Over-nourished (O)

Under-nourished (U)

Nourished (N)

- Equations need better formatting and definition. Symbols such as ℎ, Q(t), etc., should be defined immediately when first introduced.
- The transition matrix and equations (3) and (5) lack intuitive explanation. A sentence like *“This represents the instantaneous rate at which a child leaves state h and enters state j”* can aid understanding.

**Authors Response:** Thank you very much for your valuable suggestions and comments to improve our manuscript. We have carefully considered all the feedback, accepted the suggestions, and revised the manuscript accordingly as presented in both tack change and clean file at page 10.

**4. Results Presentation**

- The results are numerically rich but difficult to digest due to too many tables and minimal synthesis as suggested by reviewers

**Authors Response:** Thank you again for your insightful review. We acknowledge that the MSM output generates numerous important tables. In response to the reviewers’ suggestions, we carefully reviewed all tables and made a concerted effort to reduce redundancy. We merged several tables and moved others to the supplementary material where appropriate during first review. However, we are concerned that further reductions might compromise the clarity and completeness of the findings. We believe the current balance maintains the integrity of the results while improving readability.

**5. Interpretation and Discussion**

- **Issue**: Discussion is technically sound but lacks integration of findings into a **real-world context**.
- **Suggestions**:
  - Use subsections to structure discussion around key findings: e.g., *“Gender Differences,” “Duration in Malnutrition States,” “Impact of Maternal Education”*.
  - When discussing girls’ lower transition to undernutrition, tie findings to Ethiopian sociocultural contexts more carefully — some statements currently seem speculative (e.g., boys are prioritized but receive riskier food).
  - Add more emphasis on the **novelty of using a Markov model** and its practical implications (e.g., forecasting malnutrition trends, guiding resource allocation).

**Authors Response:** Thank you once again for your valuable suggestions. Your comments provided us with an opportunity to broaden our perspective and significantly improve the manuscript. We have carefully considered all your suggestions and revised the manuscript accordingly. Both a tracked-changes version and a clean version of the revised manuscript have been submitted. Notably, we have made substantial revisions to clarify and strengthen the novelty of the MSM at last part of discussion, strength and limitation part the study.

**6. Language and Grammar**

- **Issue**: Recurrent grammar and syntax errors, such as:

*“Girl had 1.824 times higher likelihood…”* → should be “Girls had a 1.824 times higher likelihood…”

*“Child who was older than two years of age had more likelihood…”* → should be “Children older than two years were more likely…”

**Suggestion**: A professional language edit is strongly recommended before final acceptance.

**Authors Response:** Thank you very much for your valuable suggestions and comments to improve our manuscript. We have accepted all the suggestions and made revisions accordingly. We also carefully reviewed the manuscript to correct grammatical errors to the best of our ability.

**7. Conclusion and Policy Implication**

- **Issue**: The conclusion is assertive but lacks specificity.
- **Suggestion**:
  - Offer **specific** recommendations. Instead of “enhance adult and maternal education programs,” state what kind: *“Implement targeted nutrition literacy campaigns for mothers through health extension workers.”*

**Authors Response:** Thank you very much for valuable suggestions. We carefully include all the suggestions and tried to rewrite recommendation section like**:** GO/NGO/relevant stakeholders could implement targeted literacy campaigns focus on enhancing maternal knowledge and skills related to improve child nutrition, dietary diversity, childcare, hygiene, and family planning. These campaigns should also aim to address and transform cultural beliefs and practices related to feeding habits, especially during fasting periods and to challenge harmful cultural attitudes surrounding gender roles and their impact on family health and well-being. Utilizing health extension workers or other trained health and nutrition professionals can help effectively deliver these messages at the community level. In the long run, Ministry of Health, Ministry of Agriculture, and other relevant stakeholders should strengthen efforts to reduce poverty and improve the nutritional status of children, in addition to enhancing the existing safety net programs.

==============================

Please include the following items when submitting your revised manuscript:

- A rebuttal letter that responds to each point raised by the academic editor and reviewer(s). You should upload this letter as a separate file labeled 'Response to Reviewers'.
- A marked-up copy of your manuscript that highlights changes made to the original version. You should upload this as a separate file labeled 'Revised Manuscript with Track Changes'.
- An unmarked version of your revised paper without tracked changes. You should upload this as a separate file labeled 'Manuscript'.

If you would like to make changes to your financial disclosure, please include your updated statement in your cover letter. Guidelines for resubmitting your figure files are available below the reviewer comments at the end of this letter.

If applicable, we recommend that you deposit your laboratory protocols in [protocols.io](http://protocols.io/) to enhance the reproducibility of your results. Protocols.io assigns your protocol its own identifier (DOI) so that it can be cited independently in the future. For instructions see: [https://journals.plos.org/plosone/s/submission-guidelines#loc-laboratory-protocols](https://track.editorialmanager.com/CL0/https:%2F%2Fjournals.plos.org%2Fplosone%2Fs%2Fsubmission-guidelines%23loc-laboratory-protocols/1/010f0197ab186aa4-0e1dca3f-2412-48d4-8c07-bfa209dc4143-000000/rJAwAAr6-hGqM5v1QwXuTkMmtD1dsondrOWMT1463Kg=217). Additionally, PLOS ONE offers an option for publishing peer-reviewed Lab Protocol articles, which describe protocols hosted on [protocols.io](http://protocols.io/). Read more information on sharing protocols at [https://plos.org/protocols?utm_medium=editorial-email&utm_source=authorletters&utm_campaign=protocols](https://track.editorialmanager.com/CL0/https:%2F%2Fplos.org%2Fprotocols%3Futm_medium=editorial-email%26utm_source=authorletters%26utm_campaign=protocols/1/010f0197ab186aa4-0e1dca3f-2412-48d4-8c07-bfa209dc4143-000000/iM5Cq1HeNBcnoF0FYlpH25h4ScfU9FBhKjhONRnAKOg=217).

We look forward to receiving your revised manuscript.

Kind regards,

Manzur Kader
Academic Editor
PLOS ONE

Journal Requirements:

Please review your reference list to ensure that it is complete and correct. If you have cited papers that have been retracted, please include the rationale for doing so in the manuscript text, or remove these references and replace them with relevant current references. Any changes to the reference list should be mentioned in the rebuttal letter that accompanies your revised manuscript. If you need to cite a retracted article, indicate the article’s retracted status in the References list and also include a citation and full reference for the retraction notice.


[Note: HTML markup is below. Please do not edit.]

Reviewers' comments:

Reviewer's Responses to Questions

**Comments to the Author**

1. If the authors have adequately addressed your comments raised in a previous round of review and you feel that this manuscript is now acceptable for publication, you may indicate that here to bypass the “Comments to the Author” section, enter your conflict of interest statement in the “Confidential to Editor” section, and submit your "Accept" recommendation.

Reviewer #2: (No Response)

Reviewer #3: All comments have been addressed

Reviewer #4: All comments have been addressed

2. Is the manuscript technically sound, and do the data support the conclusions?

The manuscript must describe a technically sound piece of scientific research with data that supports the conclusions. Experiments must have been conducted rigorously, with appropriate controls, replication, and sample sizes. The conclusions must be drawn appropriately based on the data presented.

Reviewer #2: Yes

Reviewer #3: Yes

Reviewer #4: Yes

3. Has the statistical analysis been performed appropriately and rigorously?

Reviewer #2: Yes

Reviewer #3: Yes

Reviewer #4: Yes

4. Have the authors made all data underlying the findings in their manuscript fully available?

The [PLOS Data policy](http://track.editorialmanager.com/CL0/http:%2F%2Fwww.plosone.org%2Fstatic%2Fpolicies.action%23sharing/1/010f0197ab186aa4-0e1dca3f-2412-48d4-8c07-bfa209dc4143-000000/iaIF9IJR2f7S__BHt8lXvlKggyCZH_bS4Lqss9rBZQk=217) requires authors to make all data underlying the findings described in their manuscript fully available without restriction, with rare exception (please refer to the Data Availability Statement in the manuscript PDF file). The data should be provided as part of the manuscript or its supporting information, or deposited to a public repository. For example, in addition to summary statistics, the data points behind means, medians and variance measures should be available. If there are restrictions on publicly sharing data—e.g. participant privacy or use of data from a third party—those must be specified.

Reviewer #2: Yes

Reviewer #3: Yes

Reviewer #4: Yes

5. Is the manuscript presented in an intelligible fashion and written in standard English?

PLOS ONE does not copyedit accepted manuscripts, so the language in submitted articles must be clear, correct, and unambiguous. Any typographical or grammatical errors should be corrected at revision, so please note any specific errors here.

Reviewer #2: Yes

Reviewer #3: Yes

Reviewer #4: Yes

6. Review Comments to the Author

Please use the space provided to explain your answers to the questions above. You may also include additional comments for the author, including concerns about dual publication, research ethics, or publication ethics. (Please upload your review as an attachment if it exceeds 20,000 characters)

**Reviewer #2:** I think the author did not answer some of the questions i asked, including :
1. In the introduction section, it is explained that Ethiopia faces high burden of under-nutrition prevalence, ranking among 15 worst affected nations, perhaps short-term or long-term targets in that field in Ethiopia can be explained as a reference. In this question the author did not answer what i asked. While the prevalence of malnutrition in Ethiopia is discussed, could comparisons to neighboring countries or regions add further perspective to the problem?

- **Author Response:** we say sorry if we did not understand or not address the questions or comments you raised in previous review. Next we have much thanks to you and appreciate your commitment to improve our manuscript. Finally, we have tried to respond it like this: Ethiopia is one of among globally affected countries but most of listed 15 countries (Afghanistan, Burkina Faso, Chad, DRC, Ethiopia, Haiti, Kenya, Madagascar, Mali, Niger, Nigeria, Somalia, South Sudan, Sudan and Yemen) were from Africa except Haiti, Afghanistan and Yemen. From the listed Kenya, South Sudan, Sudan and Somalia are neighboring countries with Ethiopia. In 2022, Ethiopia had a particularly high prevalence of stunting, at 34.4%, which is classified as "very high" according to the WHO-UNICEF Technical Advisory Group on Nutrition Monitoring thresholds established in 2018. This rate is higher than that of most neighboring countries such as Somalia (18%), Kenya (18.4%), Djibouti (18.7%), and South Sudan (27.9%)—except for Eritrea (50.2%) and Sudan (36%) (De Onis et al., 2019; UNICEF, et al, 2023). Similarly, the global prevalence of wasting among under-five children was 6.8%. In Ethiopia, the prevalence of wasting was equal to the global average at 6.8%, while the underweight rate stood at 21%. With these figures Ethiopia is better than those of most neighboring countries, with the exception of Kenya (5% wasting and 10% underweight). In terms of overweight, the global prevalence among U5C was 5.6%. Ethiopia reported a lower prevalence of 2.7%, which is below both the global average and the rates in all neighboring countries , ( EPHI & ICF, 2021; UNICEF,et al, 2023). Yes it may be short term but recurring problem, the meeting was to respond acute malnutrition which might be happened due to recurring drought, conflict, locust infestation or COVID-19 pandemic. Almost all listed countries have civil conflict in addition to the poorest economy status. The conflict and social displacement are continuing till now in Ethiopia; so the problem is short term but recurrent.

2. The authors can strengthen the practical recommendation section by elaborating on specific interventions for policymakers. For example, what types of maternal education programs would be most effective? in this question i asked the author to give an example of the concrete program

- **Authors Response:** Thank you very much for your valuable suggestions, and we apologize for any previous inconvenience. We carefully include all the suggestions and tried to rewrite recommendation section like**:** GO/NGO/ relevant stakeholder could implement targeted literacy campaigns focus on enhancing maternal knowledge and skills related to improve child nutrition, dietary diversity, childcare, hygiene, and family planning. These campaigns should also aim to address and transform cultural beliefs and practices related to feeding habits, especially during fasting periods and to challenge harmful cultural attitudes surrounding gender roles and their impact on family health and well-being. Utilizing health extension workers or other trained health and nutrition professionals can help effectively deliver these messages at the community level. In the long run, Ministry of Health, Ministry of Agriculture, and other relevant stakeholders should strengthen efforts to reduce poverty and improve the nutritional status of children, in addition to enhancing the existing safety net programs.

Reviewer #3: Thank you for the opportunity to review the revision of this manuscript. The revised version focuses on addressing critical public health challenges related to child undernutrition in Ethiopia using a continuous-time Markov multistate model. This methodological choice, combined with longitudinal data, contributes meaningfully to understanding the dynamic nature of child nutritional status transitions and the broader implications for targeted interventions.

The respected reviewer has provided thoughtful and insightful feedback, and the author has responded in a structured and reflective manner. Particularly commendable is the reviewer’s question regarding the rationale behind choosing the multistate model, which allowed the author to clarify its advantage over other statistical approaches in capturing intermediate events. Another notable point is the reviewer’s comment on gender-based differences in recovery, which encouraged the author to delve into cultural and biological factors—this reflects a strong level of critical engagement by the reviewer and a well-appreciated elaboration by the author.

While the author has addressed the reviewer’s comments diligently, a couple of aspects could be further considered. For instance, the response to the suggestion on regional comparisons could be improved with quantitative data comparing Ethiopia’s malnutrition rates to neighboring countries like Kenya or Sudan to offer clearer regional context. Additionally, while the discussion around interventions was improved, specific examples of maternal education program formats (e.g., community-based group sessions vs. mobile health interventions) would make the recommendations more actionable.

Overall, the author’s responsiveness and the reviewer’s depth of analysis are both commendable. The paper has improved significantly in clarity and relevance.

**Authors Response:** Thank you very much for your valuable suggestions. We accepted all suggestion and improved the manuscript accordingly. Ethiopia is one of among globally affected countries but most of listed 15 countries (Afghanistan, Burkina Faso, Chad, DRC, Ethiopia, Haiti, Kenya, Madagascar, Mali, Niger, Nigeria, Somalia, South Sudan, Sudan and Yemen) were from Africa except Haiti, Afghanistan and Yemen. From the listed Kenya, South Sudan, Sudan and Somalia are neighboring countries with Ethiopia. In 2022, Ethiopia had a particularly high prevalence of stunting, at 34.4%, which is classified as "very high" according to the WHO-UNICEF Technical Advisory Group on Nutrition Monitoring thresholds established in 2018. This rate is higher than that of East Africa’s average (30.6%) and most neighboring countries such as Somalia (18%), Kenya (18.4%), Djibouti (18.7%), and South Sudan (27.9%)—except for Eritrea (50.2%) and Sudan (36%) (De Onis et al., 2019; UNICEF, et al, 2023). Similarly, the global prevalence of wasting among U5C was 6.8%. In Ethiopia, the prevalence of wasting was equal to the global average at 6.8%, while the underweight rate stood at 21%. With these figures Ethiopia is better than those of most neighboring countries, with the exception of Kenya (5% wasting and 10% underweight). In terms of overweight, the global prevalence among U5C was 5.6%. Ethiopia reported a lower prevalence of 2.7%, which is below both the global average and the rates in all neighboring countries, ( EPHI & ICF, 2021; UNICEF,et al, 2023). Yea it may be short term but recurring problem, the meeting was to respond acute malnutrition which might be happened due to recurring drought, conflict, locust infestation or COVID-19 pandemic. Almost all listed countries have civil conflict in addition to the poorest economy status. The conflict and social displacement are continuing till now in Ethiopia; so the problem is short term but recurrent. We also improved conclusion part as: GO/NGOs or relevant stakeholders could implement targeted literacy campaigns either community-based group sessions or mobile health interventions which focus on enhancing maternal knowledge and skills related to improve child nutrition, dietary diversity, childcare, hygiene, and family planning. These campaigns should also aim to address and transform cultural beliefs and practices related to feeding habits, especially during fasting periods and to challenge harmful cultural attitudes surrounding gender roles and their impact on family health and well-being. Utilizing health extension workers or other trained health and nutrition professionals can help effectively deliver these messages at the community level.

Reviewer #4: The manuscript is clearly written and the study is well conceived and methodologically rigorous, offering an important addition to the area of child nutrition by using continuous-time multi-state Markov model rarely used in this area. The application of CIAF in measuring these dynamic nutritional shifts among children under the age of five in Ethiopia is particularly novel and adds practical value to the study. The authors are very responsive to reviewer comments, and much of the writing has been improved for clarity, justification and organization in key sections. Ethical approval is well recorded and data management seems to be appropriate and transparent. If published, this study will provide reasonable information for policy makers to act for malnutrition in vulnerable groups.

**Authors Response:** Thank you very much for your acceptance and kind suggestions.

7. PLOS authors have the option to publish the peer review history of their article ([what does this mean?](https://track.editorialmanager.com/CL0/https:%2F%2Fjournals.plos.org%2Fplosone%2Fs%2Feditorial-and-peer-review-process%23loc-peer-review-history/1/010f0197ab186aa4-0e1dca3f-2412-48d4-8c07-bfa209dc4143-000000/K_XzS39i9rcIGkdLMEwDOcb6sNvba_n2f08Lk9MTTuU=217)). If published, this will include your full peer review and any attached files.


If you choose “no”, your identity will remain anonymous but your review may still be made public.


**Do you want your identity to be public for this peer review?** For information about this choice, including consent withdrawal, please see our [Privacy Policy](https://track.editorialmanager.com/CL0/https:%2F%2Fwww.plos.org%2Fprivacy-policy/1/010f0197ab186aa4-0e1dca3f-2412-48d4-8c07-bfa209dc4143-000000/-ZLMMQ-F8YJBhOZNYNSwN3Er_t36RLbI4bLInpFrUzI=217).

Reviewer #2: No

Reviewer #3: **Yes:** Bushra Akter

Reviewer #4: **Yes:** Taiyeba Akter

[NOTE: If reviewer comments were submitted as an attachment file, they will be attached to this email and accessible via the submission site. Please log into your account, locate the manuscript record, and check for the action link "View Attachments". If this link does not appear, there are no attachment files.]

While revising your submission, please upload your figure files to the Preflight Analysis and Conversion Engine (PACE) digital diagnostic tool, [https://pacev2.apexcovantage.com/](https://track.editorialmanager.com/CL0/https:%2F%2Fpacev2.apexcovantage.com%2F/1/010f0197ab186aa4-0e1dca3f-2412-48d4-8c07-bfa209dc4143-000000/vthXCuYvIceRUgh4SiAfF4o4Ps6lrz6S7taR5MZegN4=217). PACE helps ensure that figures meet PLOS requirements. To use PACE, you must first register as a user. Registration is free. Then, login and navigate to the UPLOAD tab, where you will find detailed instructions on how to use the tool. If you encounter any issues or have any questions when using PACE, please email PLOS at [figures@plos.org](mailto:figures@plos.org). Please note that Supporting Information files do not need this step.
